# Plastic Pollution in Paradise: Analyzing Plastic Litter on Malta’s Beaches and Assessing the Release of Potentially Toxic Elements

**DOI:** 10.3390/toxics12080568

**Published:** 2024-08-03

**Authors:** Piotr Jachimowicz, Barbara Klik, Adriana Dorota Osińska

**Affiliations:** 1 Institute of Environmental Technology, CEET, VSB-Technical University of Ostrava, 17. Listopadu 15/2172, 708 00 Ostrava, Czech Republic; 2 Institute of Environmental Engineering, Warsaw University of Life Sciences, Nowoursynowska 159, 02-776 Warsaw, Poland; barbara_klik@sggw.edu.pl; 3 Department of Paraclinical Sciences, Norwegian University of Life Sciences, Postboks 5003, 1432 Ås, Norway; adriana.dorota.osinska@nmbu.no

**Keywords:** microplastic, mesoplastic, macroplastic, potentially toxic elements, tourism

## Abstract

This study investigates plastic litter on two beaches in Malta, Golden Bay and Rivera Beach, with a focus on plastic abundance, characteristics, sources, and the influence of human activity on pollution levels. Conducted in March 2023 during the low-tourist season, 13 sediment samples were collected from a depth of 5 cm using a systematic square sampling method. Plastic litter was quantified and sorted by size, shape, color, and polymer type, and concentrations of potentially toxic elements (PTEs) were measured (Cd, Co, Cr, Cu, Mn, Ni, Pb, Zn, and Fe via ICP-OES). Golden Bay exhibited significantly higher plastic quantities (53.9 ± 4.3 n/m^2^) compared to Rivera Beach (29.7 ± 4.0 n/m^2^). Microplastics were dominant on both beaches, with Golden Bay showing a higher proportion (57.0%) than Rivera Beach (50.6%). The plastic litter predominantly consisted of PE (59.6–68.0%) and PP (29.6–38.8%). Golden Bay plastics had PTE concentrations up to 4.9 times higher than those in Rivera Beach, notably for Mn (309.0 μg/g vs. 63.1 μg/g). This research contributes valuable insights into the dynamics of plastic pollution in coastal environments, particularly in areas influenced by tourism.

## 1. Introduction

Tourism in Malta has evolved into a significant industry over the years, with growth in tourist arrivals and substantial contributions to the country’s GDP. The popularity of this Mediterranean island destination began to soar in the 1950s, spurred by the proactive measures taken by the government [1]. Tourism, generally seen as a desirable industry, carries significant economic benefits, including high income and employment multipliers for host communities, as well as recreational and adventurous opportunities for visitors. Nevertheless, these benefits were offset by significant adverse environmental impacts [2].

Global plastic production has been experiencing exponential growth, with annual production now surpassing 400 million tons [3]. This projected increase is largely attributed to the absence of comprehensive policies aimed at discontinuing the use of single-use plastics. Consequently, plastic waste pollution has rapidly escalated into a significant global concern. Whether through intentional or unintentional actions, when plastic waste is not properly disposed of, it may end up as plastic litter in the environment, seas, and beaches. As a result, litter accumulation along the coast has become a considerable problem [4,5]. In the study conducted by Lee et al. [6], various sizes of plastic debris were observed on South Korean beaches. The abundance ranged from 8205 to 27,606 n/m^2^ for microplastics, 238 to 237 n/m^2^ for mesoplastics, and 0.97 to 1.03 n/m^2^ for macroplastics, respectively. The research revealed an exponential increase in the abundance of plastic litter as the size class decreased. This phenomenon was likely attributed to the fragmentation of large plastic litter upon reaching the shoreline. Vlachogianni et al. (2020) observed that on the beaches of southern Sicily, there were between 213 and 976 pieces of litter per 100 m of beach, of which 90% were plastic [7].

The accumulation of plastic litter along the coastline presents a significant issue, closely linked to various human activities. Furthermore, individuals who frequent recreational beaches worsen this problem by failing to dispose of their packaging, bottles, and other items properly [8]. According to observations by Nachite et al. [9], there is a noteworthy abundance of litter associated with beachgoers. These items collectively constitute a substantial 80.3% of the total litter collected.

As an island nation, Malta’s economy heavily relies on tourism, with its pristine beaches and coastal environment being major attractions. However, the increasing presence of plastic debris on beaches can deter tourists, leading to a decline in tourism revenue. Tourists often prioritize the cleanliness of beaches when making their travel decisions, along with factors like scenic beauty, safety, available facilities, and water quality. Plastic litter, in addition to its adverse environmental impacts, can lead to economic losses for local communities that rely on the financial benefits of tourism [10]. For instance, in numerous countries, the absence of litter plays a significant role in visitors’ choices, and the likelihood of returning to a particular beach is closely linked to the coastal environment’s quality [11].

Plastic litter constitutes a significant environmental threat due to its exceptional durability and resistance to biodegradation. Their surfaces have the capacity to interact with various chemical substances, including heavy metals, antibiotics, pesticides, and other pollutants, making them particularly concerning [12,13]. These potentially toxic elements (PTEs), particularly heavy metals, pose significant threats to marine life due to their non-biodegradable nature and persistent presence in the environment [14]. Major sources of PTEs include industrial emissions, agricultural runoff, shipping activities, and urban wastewater. Industrial processes often release heavy metals such as Hg, Pb, and Cd into the environment, either directly or via atmospheric deposition [15]. Additionally, shipping activities introduce PTEs through oil spills, ballast water discharge, and atmospheric emissions from vessels [16]. Marine organisms tend to accumulate these PTEs, which can lead to reduced species diversity and abundance, impacting marine ecosystems. Additionally, heavy metals contribute to fish toxicity, causing DNA damage and genotoxicity, which manifest as aberrant gene expression and DNA–protein interaction [17,18]. These contaminants can affect various levels of the food chain, from planktonic organisms to larger predators, ultimately influencing ecosystem stability and function. Ocean acidification further exacerbates these risks, potentially interacting with PTEs to pose even greater threats to marine organisms and ecosystems [19,20,21]. Jin et al. [20] suggest that the combined effects of ocean acidification and heavy metals typically exhibit additive interactions, though synergistic effects are also significant, underscoring the complexity and urgency of addressing these environmental challenges to safeguard marine biodiversity and ecosystem health. Moreover, microplastics can be inhabited by diverse microbial communities. These particles serve as vectors for the dissemination of pathogenic bacteria, harmful algae, and invasive species, thereby amplifying their adverse effects on marine ecosystems [22]. Their small size renders them especially perilous to marine life, as planktonic organisms and larger animals can ingest them [23]. Bottari et al. (2024) reported that when focusing exclusively on entanglement cases, the predominant litter type was fishing nets, accounting for 46% of the cases, followed by fishing lines at 23%, and plastic debris. Regarding the health status of the species, 74% of marine animals survived the impact of plastic waste, while 24% died [24].

The goal of this study is to address the existing research gap by conducting a comparative analysis of plastic litter in beach sediments from two different beaches in Malta, one heavily frequented by beachgoers (Golden Bay) and the other a more secluded beach (Rivera Beach), during the low-touristic season. Most research focuses on peak tourist seasons, when pollution is expected to be at its highest. By conducting this study during the low-touristic season, we can understand the baseline levels of plastic pollution and the persistence of litter from high-season activities. The study aims to assess and compare the abundance, characteristics, and sources of plastic debris at these sites, shedding light on the influence of human activity on plastic pollution. Additionally, this research will investigate the potential release of PTEs (Cd, Co, Cr, Cu, Mn, Ni, Pb, Zn, and Fe) from the collected plastics, providing insights into the environmental implications of hazardous chemicals associated with plastic debris. Through these investigations, we aim to contribute to a better understanding of the complex interplay between tourism and plastic litter in coastal ecosystems.

## 2. Materials and Methods

### 2.1. Study Area

**Figure 1:**
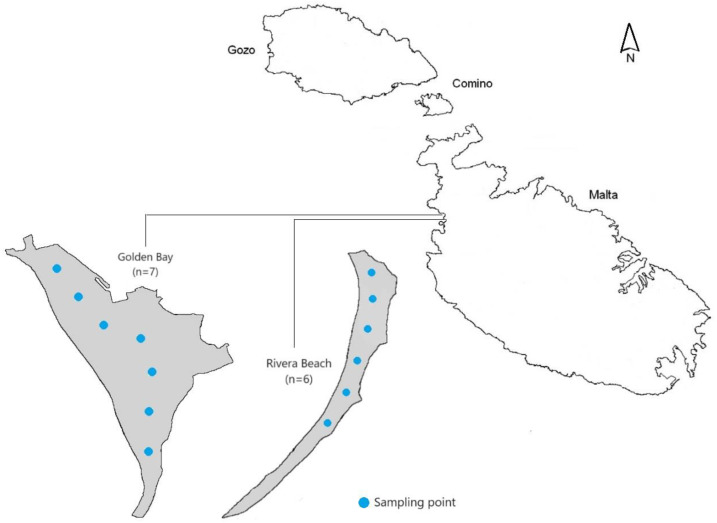
The locations of sampling points at the beach on Malta Island.

This study was conducted at two beaches in Malta: Golden Bay and Rivera Beach (Figure 1). Malta is an archipelago located in the Mediterranean Sea, consisting of several islands, with Golden Bay and Rivera Beach situated on the main island of Malta. Golden Bay, with approximately 220 m of shoreline, features a gently sloping beach with fine sand. On the other hand, Rivera Beach, which extends over 315 m, shares characteristics similar to Golden Bay, making it a relevant comparison site for assessing plastic pollution. The main difference is that Golden Bay is classified as a typical tourist beach with developed infrastructure and nearby hotels. In contrast, Rivera Beach is primarily used by locals [25].

### 2.2. Sampling

In March 2023, samples were gathered during Malta’s low-tourist season, when there was no legal requirement for beach cleaning. A total of 13 samples were collected for analysis from Golden Bay (*n* = 7) and from Rivera Beach (*n* = 6), utilizing the square method (Figure 1). Each m^2^ was delineated using cotton thread and wooden stakes. These squares were positioned approximately 25 m apart. Sampling points were strategically located in the middle of the beach width to minimize the potential impact of factors like plastic transportation by waves or wind. Sediment samples were taken from a depth of 5 cm. At Rivera Beach, the number of sampling points was reduced due to a notable decrease in beach width caused by cliffs and its transformation into rockier terrain.

The sieved plastic litter (>0.5 mm) was subsequently transported to the laboratory, where it underwent a gentle wash in distilled water and was then dried at room temperature using airflow before undergoing further analysis.

### 2.3. Sample Processing

Plastic debris was quantified and sorted at all sampling points based on size, shape, color, and polymer type. To assess the size distribution of plastic litter, we classified them into three size classes: microplastics (0.5 to <5 mm), mesoplastics (5 to <25 mm), and macroplastics (≥25 mm), according to González et al. [26]. Particles smaller than 0.5 mm could not be identified or counted without microscopic observation and subsequent spectroscopic confirmation. Therefore, our study focused on microplastics in the size range of 0.5–5 mm. Plastic litter shapes, both visually and microscopically, are classified as fibers (including filaments and fishing lines), fragments (items with a three-dimensional shape), and pellets (solid spheres). The abundance of different colors of plastics was classified into 11 color classes, including white, red, orange, blue, black, grey, brown, pink, green, yellow, and purple.

To determine the polymer type of the collected plastic items, we used Fourier-transform infrared (FTIR) spectroscopy. The analysis was performed using Shimadzu IR Spirit (Kyoto, Japan), equipped with a single reflection diamond Attenuated Total Reflectance (ATR) with spectra measured in the wavelength range of 400–4000 cm^−1^ with a resolution of 4 cm^−1^. The obtained spectra were correlated with the reference spectra of the software library to identify the types of polymers. In the present study, only spectra with 70% similarities to the reference spectra were accepted. Based on the concentration and chemical composition of different polymer types of MPs on beaches, the probable chemical risks were assessed through Polymer Hazard Index (PHI) values. This index takes into account the percentage of specific polymer types collected at each sampling location and the hazard scores of these polymer types [27].

### 2.4. Potentially Toxic Elements

The plastic litter samples from Golden Bay and Rivera Beach were meticulously washed in ultra-pure water. Subsequently, they were air-dried, thoroughly mixed, and finely shredded into particles of 1–2 mm in size. The prepared samples underwent digestion in HNO_3_ and H_2_O_2_ (4:1) in Teflon vessels using a microwave oven (MARSXpress, CEM Corporation, Matthews, NC, USA). After digestion, the concentrations of Cd, Co, Cr, Cu, Mn, Ni, Pb, Zn, and Fe were determined through optical emission spectroscopy with inductively coupled plasma (ICP-OES) (iCAP PRO Series, Thermo Scientific™, Waltham, MA, USA) [28,29]. All analyses were conducted in triplicate to ensure precision and reliability. For calibration, multielement TraceCERT^®^ standards for ICP (Sigma-Aldrich, Dorset, UK) were utilized. The effectiveness of the analysis was verified using Certified Reference Material (CRM 142 R). The values for LOD (limit of detection), LOQ (limit of quantification), and accuracy (AO) for the analyzed elements are presented in Table 1.

### 2.5. Statistical Analysis

An analysis of variance (ANOVA) was conducted to determine the differences among individual sampling sites in terms of plastic litter concentrations as well as its distribution by shape, size, and color. To identify significant pairwise differences between the sampling sites, we used the Tukey Honest Significant Differences (HSD) post-hoc test. The significance level was set at *p* < 0.05. Additionally, Pearson correlation analysis was performed to examine the relationship between plastic litter abundance and the number of beachgoers. All statistical analyses were conducted using Statistica 13.3 (StatSoft, Tulsa, OK, USA).

## 3. Results and Discussion

### 3.1. Variations in Plastic Litter Quantities

**Figure 2:**
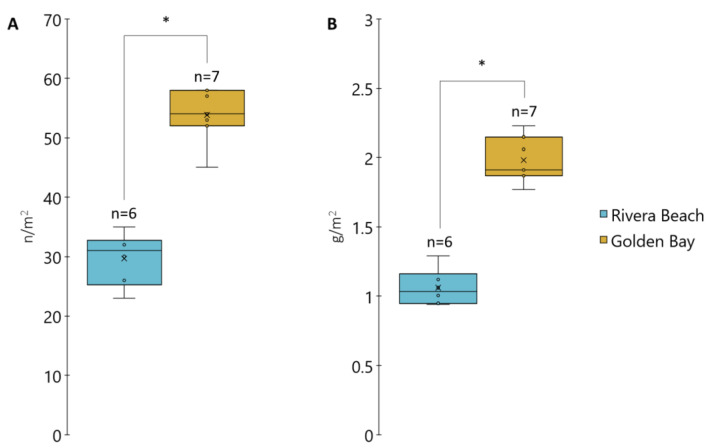
The average number (**A**) and mass (**B**) of collected plastic litter at the beaches (* significantly different at *p* < 0.05).

The quantity and weight of plastic litter exhibited significant variations between the beaches (Figure 2). The total number of plastic particles collected at Golden Bay was 377, while Rivera Beach had a total of 178 plastic particles. Golden Bay demonstrated significantly higher quantities of plastic litter (*p* < 0.05), with an average of 53.9 ± 4.3 n/m^2^, compared to Rivera Beach, where the abundance was 29.7 ± 4.0 n/m^2^. The actual pollution from plastic litter could be significantly greater than stated above, as our study did not analyze small microplastics (<0.5 mm). In a study conducted by Constant et al. [30], plastic particles with a diameter of less than 0.5 mm accounted for 52.3–55.5% of all collected plastic litter from the beach. The results indicate that Rivera Beach, with a PHI of 1.021, poses a relatively medium environmental and health risk due to the predominance of lower-hazard-score plastics like PP and PE. In contrast, Golden Beach has a higher PHI of 1.118, reflecting a greater potential hazard from the presence of higher-hazard-score plastics such as PVC, PET, and PS.

Furthermore, the quantity of plastic litter correlated with the total mass of plastics found on the beach. The recorded weights were 1.06 ± 0.12 g/m^2^ for Rivera Beach and 1.98 ± 0.15 g/m^2^ for Golden Bay. Axiak et al. [31] also reported significantly higher concentrations of plastic litter, particularly microplastics, in Golden Bay compared to Rivera Beach in their earlier research. They suggested that this disparity might be attributed to the proximity of Golden Bay to commercial establishments, such as hotels and restaurants, as well as the prevalence of recreational activities like water sports.

**Figure 3:**
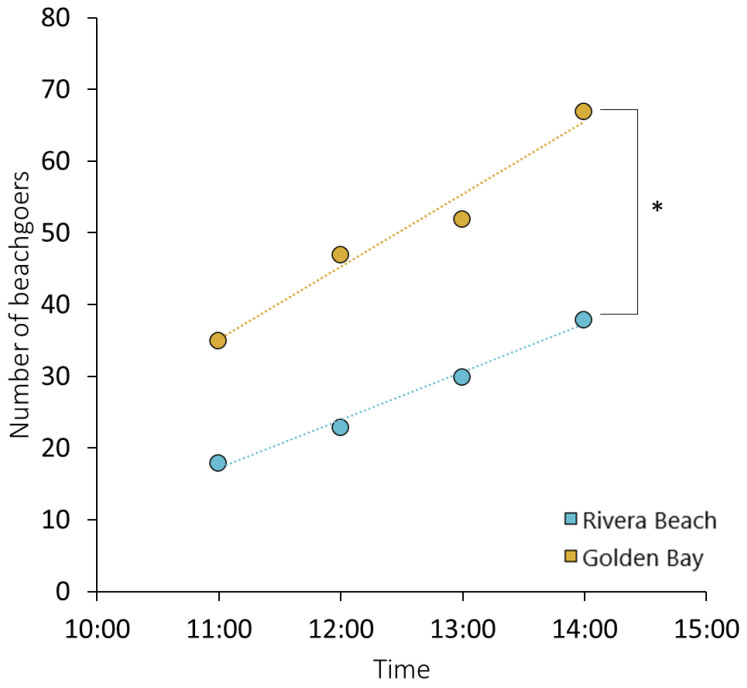
Number of beachgoers at beaches (* significantly different at *p* < 0.05).

In conclusion, our study found a significant correlation (*p* < 0.05) between the concentrations of plastic litter and the accumulated mass on the beach surface, related to the number of beachgoers (Figure 3). Beach visitors, coastal inhabitants, and recreational activities are the major contributors to plastic pollution due to their behavior and plastic usage [32].

### 3.2. Dominance of Microplastic and Debris Breakdown

**Figure 4:**
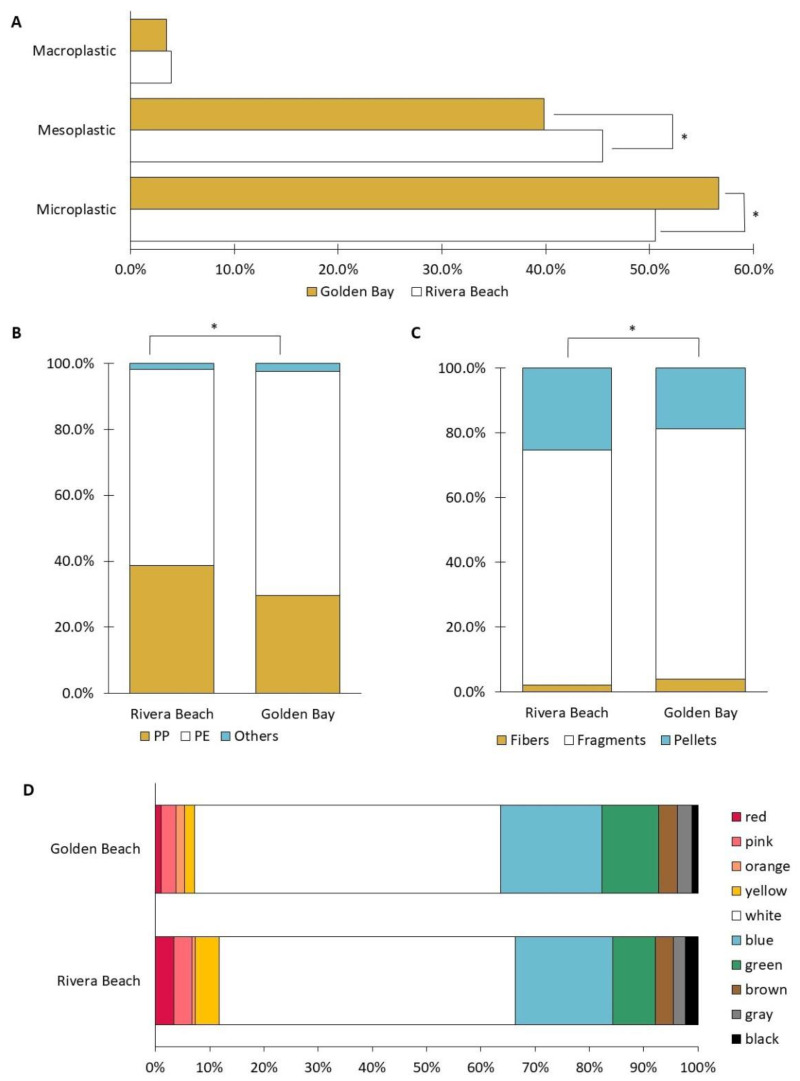
Composition of size (**A**), polymer type (**B**), shape (**C**), and color (**D**) of collected plastic litter (* significantly different at *p* < 0.05).

The analysis of plastic litter sizes at both beaches revealed that microplastics constituted the most abundant category, followed by mesoplastics and macroplastics (Figure 4A). In the case of both beaches, microplastics accounted for more than 50% of all the collected plastic litter. However, it is noteworthy that in Golden Bay, their proportion was significantly higher than in Rivera Beach (57.0% vs. 50.6%). Conversely, for mesoplastics, significantly higher proportions were observed in Rivera Beach (45.5% vs. 40.0%). Our results are consistent with those of other researchers, where over 90% of beach-collected plastic litter consisted of microplastics and mesoplastics [33].

In our study, no significant differences were detected between the two beaches regarding macroplastics, and their contribution remained below 4.0%. This trend was accompanied by a decrease in the size of plastic fragments as the abundance of plastic debris increased. Barnes et al. [34] documented a general reduction in the average size of plastic debris in the global environment, which correlated with an increasing abundance of these particles due to continuous degradation processes.

Moreover, the ratio of microplastics to mesoplastics is notably higher on Golden Bay Beach, indicating a considerably swifter breakdown of plastic litter, posing an increased threat to the aquatic environment. Beaches provide favorable conditions for the disintegration of plastic debris due to the effects of weathering degradation, including wind and wave action [35]. Consequently, there is a high probability that plastic debris present on beaches will continue to fragment into smaller particles.

### 3.3. Supremacy of PE and PP on the Beaches of Malta

The results of the chemical analyses reveal limited diversity in the types of plastic litter on the beaches (Figure 4B). PE emerged as the dominant polymer, accounting for 59.6–68.0% of the particles. Following closely, PP constituted the second most prevalent polymer, making up 29.6–38.8% of the particles. Additionally, polymers such as PET, PS, and PU were also detected; however, their combined presence did not exceed 2.5%. A study conducted by Expósito et al. [36] along the western Mediterranean Sea beaches similarly observed a predominance of PE and PP among the collected plastic particles.

Significant differences in polymer composition are noticeable between the beaches. In Golden Bay, there is notably more PE (68.0% vs. 59.6%), while in Rivera Beach, there is a higher proportion of PP (38.8% vs. 29.6%). The sources of these polymers found on the beach were primarily attributed to the breakdown of larger objects. PP is often used to make packaging, bottle caps, ropes, and drinking straws, while PE is used in supermarket bags and plastic bottles [37]. Both types of polymers (PP and PE) are plastic resins with a specific gravity of less than one, permitting them to be positively buoyant and easily deposited on beaches [35].

The PE/PP ratio was also calculated, resulting in values of 2.29 and 1.53 for Golden Bay and Rivera Beach, respectively. These findings indicated that a high PE/PP ratio in Golden Bay suggested a preferential accumulation of more aged fragments on the beach [38]. However, the overall PE/PP ratios reflect the relative production rates for various polymer types, with PE being the most produced polymer globally [3].

### 3.4. Composition of Plastic Litter Shapes

Plastic litter detected in samples from Malta’s beaches was classified into three main shapes: fragments, fibers, and pellets (Figure 4C). Fragments dominated in both Golden Bay and Rivera Beach, constituting 77.3% and 72.5% of the total collected plastic litter, respectively. In both areas, fragments dominated over pellets, accounting for 18.7% and 25.3%, respectively. The abundance of fibers in samples was less than 4%.

The breakdown of larger objects into smaller fragments primarily occurs due to processes such as photooxidation, thermal degradation, and biodegradation [35]. However, the rates and mechanisms of fragmentation may differ depending on the type of polymer. For instance, PE tends to fragment more readily due to weathering events, while PP is more susceptible to mechanical degradation [39]. In our study, we observed a variety of shapes among the fragments, with a majority being irregular, jagged pieces originating from larger plastic items.

A higher percentage of plastic pellets was observed in Rivera Beach compared to Golden Bay. According to the findings presented by Turner and Holmes [40], the exact sources of plastic pellets along the Maltese coastline remained undisclosed. Plastic pellets are typically associated with industrial areas, and in this particular instance, they were situated to the south of Malta. Southeasterly currents served as a significant impediment, hindering their transportation to the beaches. Another reason for the greater proportion of pellets in beach samples is likely the direct loss of plastic pre-production pellets during transportation by plastic manufacturing industries [41].

The presence of fibers in the analyzed samples was minimal. Typically, fibers constitute a significant portion of the entire plastic litter assemblage, with their primary source being fishing nets [42]. Consistent with the findings of Edo et al. [38], where the proportion of fibers was below 0.2%, our study indicates that in such cases, the presence of microplastics signifies a form of diffuse pollution typical of remote areas distant from major sources of human activity. This underscores the global dispersal capability of microplastics.

### 3.5. Color Distribution and Ecological Insights

The predominant colors of plastic litter found on the beaches were white (54.5–56.5%), blue (18.0–18.7%), and green (7.9–10.4%) (Figure 4D). Similar trends in color prevalence have been observed in the work of other researchers, emphasizing the dominance of white-colored plastic debris [43]. The prevalence of white plastic litter in our study can be attributed to the beach environment, as most beach equipment, including sun loungers and items brought by tourists, is often white. Noticeable discoloration was observed in the collected plastic debris, primarily due to the effects of weathering [44].

In this study, colorful plastic debris in red, pink, yellow, orange, brown, gray, and black colors was also identified, yet their combined representation did not exceed 20%. Research by Liu et al. [45] suggests that photochemical processes involving inorganic pigments in colored plastic debris may result in the release of heavy metals. The color of plastic debris is important in a biological context, as an organism’s feeding preferences may be influenced by the color of plastic objects, making them more or less likely to be mistaken for food [46].

### 3.6. Potentially Toxic Element Contents

**Figure 5:**
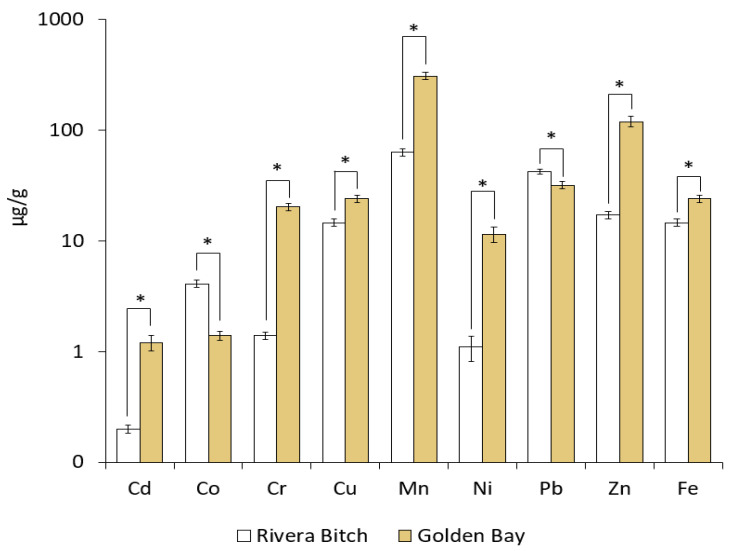
PTE concentration in plastic litter at the beaches of Golden Bay and Rivera Beach (* significantly different at *p* < 0.05).

The analysis of plastic debris collected from Golden Bay and Rivera Beach revealed significant differences in the concentrations of toxic elements. Golden Bay, a tourist beach with developed infrastructure located on the main island of Malta, exhibited significantly higher concentrations of the most investigated PTEs compared to the local Rivera Beach (Figure 5).

In the case of Cd, the concentration in Golden Bay was significantly higher (1.2 μg/g) than in Rivera Beach (0.2 μg/g), suggesting greater plastic pollution on the tourist beach compared to the beach primarily utilized by the local community. Similar trends were observed for Cr, Cu, Ni, Zn, and Fe, where concentrations were higher in Golden Bay than in Rivera Beach. The most significant difference was observed for Mn, with the concentration in Golden Bay (309.0 μg/g) significantly higher than in Rivera Beach (63.1 μg/g), indicating considerably higher plastic contamination, approximately 4.9 times higher on the tourist beach. This could be attributed to the previously mentioned higher accumulation of plastic litter in Golden Bay compared to Rivera Beach, induced by changes in the chemical and physical properties of plastic litter caused by weathering [47]. Weathering has the potential to enhance the mobility of chemically non-bound compounds in plastic litter, like metalloids [48]. Similar trends were observed by Carbery et al. [49], who also confirmed that plastic collected on Australian beaches showed the highest concentrations of Mn > Zn > Pb. Compounds of metalloids are added to plastics as fillers, heat and UV stabilizers, color pigments, release agents, activators, biostabilizers, antimicrobial agents, catalysts, and intermediates. In expanded and extruded materials, especially rigid polyurethane, some compounds of Sb and Zn also serve as flame retardants and/or smoke suppressants [50].

Interestingly, Pb and Co exhibited divergent trends between Rivera Beach and Golden Bay, with Rivera Beach recording higher Pb levels at 42.2 μg/g and higher Co levels at 4.1 μg/g compared to Golden Bay (approximately 1.3 times and 2.9 times higher, respectively). This correlates with the observation of a greater quantity of plastic in red and yellow colors on Rivera Beach compared to Golden Bay (Figure 4D). According to the studies by Filelli and Turner [51], the presence of Pb in plastic may result from the use of various pigments containing this metal, such as red lead (Pb_3_O_4_), chrome yellow (PbCrO_4_), and cremnitz white ((PbCO_3_)_2_·Pb(OH)_2_), which are used to achieve various colors in plastics. Given their bioaccumulative potential, persistence, high toxicity, and widespread availability, PTEs sorbed into plastic could leach into aquatic ecosystems through direct ingestion of metal-laden microplastics, becoming bioavailable to freshwater and marine biota. This, in turn, leads to the transfer of these contaminants into the food chain, where they can accumulate and biomagnify across trophic levels [15,16].

## 4. Conclusions

Research provides valuable insights into the characteristics of plastic litter on the beaches of Golden Bay and Rivera Beach in Malta. The study revealed significant variations in the quantity and composition of plastic litter between these two beaches. Golden Bay exhibited higher quantities of plastic litter, with microplastics being the predominant category. Conversely, mesoplastics were more abundant in Rivera Beach. This suggests that Golden Bay may face a more substantial plastic pollution issue. PE and PP emerged as the predominant polymers on both beaches. Golden Bay Beach exhibits significantly higher concentrations of PTEs compared to Rivera Beach, particularly Mn, which is approximately 4.9 times higher. Additionally, Pb and Co levels are higher in Rivera Beach, suggesting a localized source of contamination possibly related to the presence of red and yellow plastic. Our study contributes to the understanding of plastic pollution in coastal environments and emphasizes the need for comprehensive monitoring and management strategies to mitigate the environmental impact of plastic litter on these beaches.

To address the limitations of this research, future studies should include an analysis of smaller particles, extending below the 0.5 mm threshold. This expanded analysis will provide a more comprehensive understanding of microplastic pollution, including the identification and quantification of finer particles.

## Figures and Tables

**Table 1:** LOD, LOQ, and accuracy for the investigated metals.

Elements	LOD (mg/L)	LOQ (mg/L)	Reference Value (mg/kg)	Measured Value (mg/kg)	AO (%)
Cd	0.023	0.075	14.0 ± 1.4	13.5 ± 1.6	96.4
Co	0.046	0.063	66.5 ± 2.3	67.2 ± 1.9	101.1
Cr	0.072	0.219	138.0 ± 5.0	133 ± 3.1	96.4
Cu	0.008	0.027	46.9 ± 1.8	44.9 ± 2.3	95.7
Mn	0.061	0.176	112.0 ± 6.4	113.3 ± 4.3	101.2
Ni	0.056	0.171	94.0 ± 5.0	91.8 ± 1.7	97.7
Pb	0.020	0.060	51.3 ± 2.0	49.6 ± 2.4	96.7
Zn	0.066	0.220	270.0 ± 8.0	261.9 ± 6.5	97.0
Fe	0.051	0.098	320.0 ± 11.4	318.6 ± 8.7	99.6

## Data Availability

Data are contained within the article.
